# Meta-analysis of breast cancer microarray studies in conjunction with conserved *cis*-elements suggest patterns for coordinate regulation

**DOI:** 10.1186/1471-2105-9-63

**Published:** 2008-01-28

**Authors:** David D Smith, Pål Sætrom, Ola Snøve, Cathryn Lundberg, Guillermo E Rivas, Carlotta Glackin, Garrett P Larson

**Affiliations:** 1 Division of Information Sciences, City of Hope National Medical Center, Duarte, CA 91010, USA; 2 Department o f Molecular Biology, City of Hope and Beckman Research Institute, Duarte, CA 91010, USA; 3 Division of Molecular Medicine, City of Hope and Beckman Research Institute, Duarte, CA 91010, USA; 4 Department of Computer and Information Science, Norwegian University of Science and Technology, NO-7489 Trondheim, Norway; 5 Department of Cancer Research and Molecular Medicine, Norwegian University of Science and Technology, NO-7489 Trondheim, Norway

## Abstract

**Background:**

Gene expression measurements from breast cancer (BrCa) tumors are established clinical predictive tools to identify tumor subtypes, identify patients showing poor/good prognosis, and identify patients likely to have disease recurrence. However, diverse breast cancer datasets in conjunction with diagnostic clinical arrays show little overlap in the sets of genes identified. One approach to identify a set of consistently dysregulated candidate genes in these tumors is to employ meta-analysis of multiple independent microarray datasets. This allows one to compare expression data from a diverse collection of breast tumor array datasets generated on either cDNA or oligonucleotide arrays.

**Results:**

We gathered expression data from 9 published microarray studies examining estrogen receptor positive (ER+) and estrogen receptor negative (ER-) BrCa tumor cases from the Oncomine database. We performed a meta-analysis and identified genes that were universally up or down regulated with respect to ER+ versus ER- tumor status. We surveyed both the proximal promoter and 3' untranslated regions (3'UTR) of our top-ranking genes in each expression group to test whether common sequence elements may contribute to the observed expression patterns. Utilizing a combination of known transcription factor binding sites (TFBS), evolutionarily conserved mammalian promoter and 3'UTR motifs, and microRNA (miRNA) seed sequences, we identified numerous motifs that were disproportionately represented between the two gene classes suggesting a common regulatory network for the observed gene expression patterns.

**Conclusion:**

Some of the genes we identified distinguish key transcripts previously seen in array studies, while others are newly defined. Many of the genes identified as overexpressed in ER- tumors were previously identified as expression markers for neoplastic transformation in multiple human cancers. Moreover, our motif analysis identified a collection of specific *cis*-acting target sites which may collectively play a role in the differential gene expression patterns observed in ER+ versus ER- breast cancer tumors. Importantly, the gene sets and associated DNA motifs provide a starting point with which to explore the mechanistic basis for the observed expression patterns in breast tumors.

## Background

Variation in gene expression provides a quantifiable trait that has been employed to classify breast tumors [1-3]. However it has long been known that the gene sets identified from independent laboratories fail to provide a unified set of genes thereby casting doubt on the biological implications of these profiles [4]. Despite these differences, two prognostic tests have recently been approved in the United States for clinical management of disease [5,6]. From a diagnostic perspective, developing a unified gene profile that predicts both risk of recurrence and therapeutic response in diverse disease subtypes would be clinically useful. These gene sets could also provide an understanding of the mechanistic basis of malignancy.

Meta-analysis has been used as a formal summarization method in the clinical cancer literature for many years [7-10]. Recently, some groups have applied meta-analysis to gene expression microarrays [11-13]. Meta-analysis refers to a broad class of models used for summarizing and synthesizing studies to estimate their overall effect. Rhoades, et al was among the first to demonstrate the usefulness of meta-analytic procedures on microarray data in prostate cancer [14]. Since then, there have been many contributions to the oncology literature by applying meta-analysis to microarrays, including breast cancer [13,16,17].

One of the central goals in gene expression experiments is to identify the common regulatory themes and *cis*-elements responsible for the observed patterns of gene expression. This has been most successfully performed for the yeast *Saccharomyces cerevisiae *where new regulatory genes have been suggested [18]. However, metazoan expression patterns tend to be more complicated. One approach has been to combine expression data of orthologous genes from diverse organisms to build co-expression networks [19]. In *Drosophilia *gene networks have been proposed based upon the co-localization of TFBS with *cis*-regulatory modules (CRM) [20]. The availability of both mammalian and lower metazoan complete genome assemblies affords one the opportunity to identify phylogenetically conserved motifs in the array candidates. In addition to known TFBS, these phylogenetic motifs may identify important new *cis*-acting signals that modulate transcription (promoters) or transcript stability (3'UTRs) and may be key elements in the observed expression patterns. A systematic comparison of both known and phylogenetic *cis*-elements between two sets of differentially expressed genes can serve to implicate these elements as common modulators in the observed gene expression patterns.

Our method incorporates a meta-analysis model to rank genes into groups of over- and under-expressed gene sets, based upon their relative importance between independent array studies. Our analyses of gene expression patterns in ER+ and ER- breast tumors were performed across different array platforms on a diverse spectrum of patients. The two sets of genes showing the most disparate expression patterns between ER+ and ER- tumors provided an entry point with which to explore the possibility that specific sequence elements may be disproportionately represented in these two groups. We utilized known motifs in conjunction with comparative genomic resources to search for enriched DNA elements in both the proximal promoter and 3'UTR regions of these genes. Our findings suggest that the differential gene expression in ER+ vs. ER- tumors may, in some cases, be mediated by specific sequence elements in either the promoter or 3'UTR intervals. The motif distribution profiles between our gene sets identified both known and phylogenetically conserved elements that may play a role in these genes' co-expression.

## Results

Forty-six percent of unique probes among the studies mapped many-to-one to unique UniGene IDs. The mean and median numbers of probes per UniGene IDs were 12.7 and 1, respectively. When we merged the 9 studies in Table 1 for the meta-analysis data set, we retained the expression values for all probe combinations in all studies and this resulted in a multiplicative set of records in the database. Approximately 12% of the unique ESTs in the Oncomine database (Oncomine DB) did not correspond to a unique UniGene ID. These were dropped from the analysis data sets.

**Table 1:** Breast Cancer Gene Expression Datasets used in Meta-Analysis

**Author**	**Journal**	**Array Type, N Probes**	**Sample N ER+**	**Sample N ER-**	**Other Relevant Clinical Criteria**
Wang, Y. et al.	Lancet [73]	Affy, 22283	209	77	DFS 5 Yr
Zhao, H. et al.	Mol Biol Cell [80]	cDNA, 27276	24	11	PR Status, Grade, HER2, LN Status
Sotiriou, C. et al.	PNAS [81]	cDNA, 7549	65	34	LN Status, Chemo/Radio/Horm Tx, 5 Yr OS
Ma, X. et al.	PNAS [82]	cDNA, 1940	18	5	PR, Grade, HER2, Grade, Histology
Van de Vijver, M. et al.	NEJM [83]	cDNA, 23130	226	69	DFS 5 Yr, LN Status, T/M Stage
Gruvberger, S. et al.	Ca Res [84]	cDNA, 3369	28	30	
Sorlie, T. et al.	PNAS [2]	cDNA, 7937	56	18	DFS 5 Yr, LN Status, M Stage
West, M. et al.	PNAS [85]	Affy, 6718	25	24	
Perou, C. et al.	PNAS [1]	cDNA, 8838	26	9	Before/After Chemo, Histology, Grade

DFS, Disease Free Survival; Pr, Progesterone status; LN, Lymph node status; OS, overall survival

**Figure 1:**
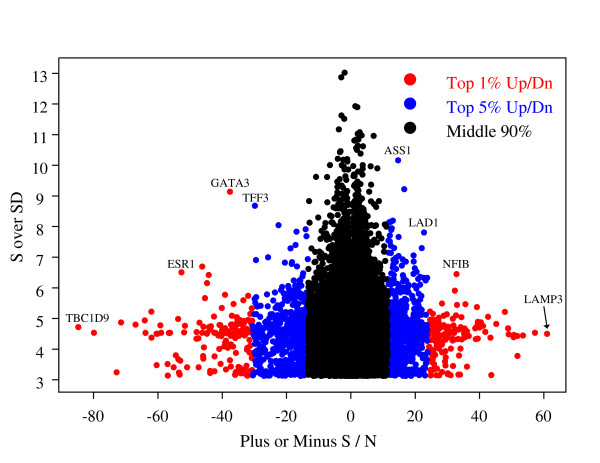
**Plot of S over N versus S over standard deviation for all genes across all studies in the meta-analysis**. Some genes present in ER+ overexpressed tumors (*ESR1 *and *GATA3*) and ER- overexpressed tumors (*LAD1 *and *NFIB*) are indicated. The top 5% of genes also include the top 1% of genes.

We focused our subsequent analyses on a select set of genes by taking medians across each UniGene ID's *S*/*SD *statistics. A scatter plot of the *S*/*N *(x-axis) versus abs(*S*)/*SD *statistics (y axis) appears in Figure 1. The distribution of the *S*/*N *values was bell-shaped with heavy tails. Our criteria for selecting genes were to take the most extreme 1% and 5% values in both tails. We found it instructive to consider the ratio *S*/*SD *on the y-axis of Figure 1, where *SD *is the standard deviation of the (*C*_*j *_ln *p*_*j*_) addends of *S*. Large values of this ratio indicate those genes with consistently significant p-values across all of the studies that we considered. The number of UniGene IDs with *S*/*N *scores in the top 1% and 5% (*S*+ and *S*- combined) were 300 and 1804, respectively. The mean numbers of studies for genes present in the top 1% and top 5% classes were 2.94 and 3.18 respectively. Our choice of reporting both top 1% and the top 5% for further screening was for crude management of false positives from bias correlated with each gene's relative ranking. Many of the genes present in our top 1% upregulated list identified in our meta-analyses have previously been identified as overexpressed in ER+ breast tumors, most notably the two transcription factors *ESR1 *and *GATA3*. Our gene lists appear in Additional File 1.

We next compared our top 1% and 5% upregulated gene lists in ER+ and ER- tumors to those prognostic genes utilized in the 70-gene signature associated with Mammaprint^® ^[21] along with the 16-gene signature with the RT-PCR based OncoType Dx^® ^[22] tests. Although the array data defining the 70-gene profile was one of 9 input datasets for our meta-analysis and the 16-gene signature datasets utilized two expression datasets were also employed for the analysis, we did not observe complete overlap in the genes identified. For the 70-gene signature our top 1% dataset identified an overlap of one and four genes respectively that were upregulated in ER+ tumors versus ER- tumors. Only 14 and 5 genes overlapped in the top 5% dataset, respectively. Alternatively, for the 16-gene signature, one and two genes, respectfully, from the top 1% gene sets were overexpressed in ER+ versus ER- tumors from our meta-analysis, while 4 and 5 genes, respectfully, overlapped in the top 5% list. Differences in probes, arrays, and studies used in the meta-analyses may explain some of the differences between our gene lists and the gene lists from the two diagnostic tools. Additionally, we compared our gene lists to a previously identified universal profile that uses 69 genes overexpressed in a diverse spectrum undifferentiated cancers to predict neoplastic transformation [23]. Strikingly we observed only genes overexpressed from ER- tumors to overlap with this 69 gene signature. Four genes (*CNAP1*, *CDC20*, *YBX1*, and *CENPA*) overlapped in our top 1% list while 23 genes overlapped from our top 5% list. These findings are in accord with the observation that ER- tumors are more highly undifferentiated than ER+ tumors and demonstrate more metastatic potential clinically [24,25]. Collectively these 23 genes may identify a set of candidate genes predictive of metastatic potential in ER- breast tumors.

### Ingenuity Pathway Analyses

We considered the relationship of our top 1% genes in the ER+ and ER- groups using Ingenuity Pathway Analyses [26]. Our objective in using Ingenuity was to characterize the functional role of our selected genes. IPA isolated genes for which it had documented associations, and created a series of networks based on the published literature. We were able to map 290 of the 300 genes comprising the sum of the 1% upregulated and 1% downregulated gene sets. From these networks, IPA queried its database of biological functions and scored each gene cluster with a p-value calculation. Table 2 shows the most common functions found among our most differentially-expressed genes. Notably our top 1% genes upregulated in ER- tumors contained 26 genes showing association to cancer whereas only 7 of the genes upregulated in ER+ tumors were cancer-associated.

**Table 2:** Ingenuity functional roles among the top 1% ER+ and ER- upregulated genes.

Function	N Genes	Ingenuity p-value
**Top 1% ER+ Upregulated**		
Small Molecule Biochemistry	16	7.71E-07
Molecular Transport	10	2.03E-04
Nervous System Development and Function	10	5.89E-03
Lipid Metabolism	9	7.71E-07
Cancer	7	5.89E-03
**Top 1% ER- Upregulated**		
Cancer	26	4.08E-04
Cellular Growth and Proliferation	23	3.34E-07
Cell Death	22	9.14E-08
Tissue Morphology	19	3.60E-05
Hematological System Development and Function	18	6.73E-06

### Promoter Motif Comparisons in Dysregulated Genes

We tested the hypothesis that there was a significant difference in the occurrence of each motif between our two classes of genes (ER+ overexpressed vs. ER- overexpressed) using a Fisher's Exact test. We adjusted for multiple testing by applying the Benjamini-Hochberg p-value correction [27]. We counted the number of genes in each class which were overexpressed in ER+ tumors and contained a copy of each phylogenetic motif, and compared those to the number of genes overexpressed in ER- tumors. For genes harboring multiple copies of a motif we counted these elements as a single motif event. We independently performed tests for both the top 1% and 5% of our genes. Our initial query sets consisted of 123 condensed TRANSFAC motifs and a second analysis comprised 174 phylogenetically conserved mammalian promoter motifs as previously defined [28]. Sixty-nine of the phylogenetic motifs map to known TFBS defined in the TRANSFAC DB v7.4 while 105 represented novel phylogenetically conserved elements.

We first examined whether any of 123 known TFBS were disproportionately represented in our ER+ and ER- gene sets. Abbreviated results appear in Table 3. While numerous motifs showed significance by Fisher's Exact testing (p < 0.05) only 2 survived multiple testing correction. The first motif KTWGTTT, a binding site for the SRY1 transcription factor, was over-represented in the top 5% of ER+ upregulated genes in the noncoding strand. For ER+ overexpressed genes 473 of 735 genes contained the site while 423 of 766 ER- overexpressed genes contained the site (Benjamini-Hochberg corrected p = 0.042). The second site, ABWCAGGTRNR, a binding site for AREB6 (also called Transcription Factor 8, TCF8, or ZEB1), was over-represented in the top 1% of ER+ upregulated genes when both coding and noncoding strands were surveyed (adjusted p = 0.024) and contains an embedded E-box motif. Twenty-five genes bore TCF8 sites in either strand amongst 138 ER+ upregulated genes while only 6 genes contained the site amongst 147 ER- upregulated genes. The presence of TCF8 sites in nearly four times as many ER+ upregulated genes versus ER- upregulated genes may be an indirect mechanism for gene activation in ER+ breast tumors. TCF8 has been shown to be induced by estrogen which in turn activates a cascade of downstream genes [29]. Additionally, the transcriptional repression of e-cadherin by TCF8 has been shown to lead to loss of the epithelial phenotype suggesting a role for this TF in late-stage carcinogenesis [30]. We note that although e-cadherin was not identified in our meta-analysis, 2 related genes, *CDH3 *and *PCDH8*, both of which lie in the top 5% of ER- overexpressed genes, may be responsive to repression by TCF8. The over-representation of TCF8 binding sites in both strands of our top 1% genes ER+ overexpressed tumors suggests that TCF8 may act as a transcriptional activator for these genes yet act as a transcriptional repressor in ER- overexpressed genes.

**Table 3:** Top Scoring Promoter TFBS Motifs Identified in Coding and Non-coding Strands. Top 1% and 5% Gene Sets.

**Rank**	**Known Motif**	**Factor**	**Genes with Motif**	**Genes w/o Motif**	**Genes with Motif**	**Genes w/o Motif**	**Fisher Raw p-value**	**Hochberg adj p-value**
			**Genes Overexpressed in ER+ Tumors**		**Genes Overexpressed in ER- Tumors**			

	*Top 1% Known Motifs Coding Strand*							
1	CTTTGA	LEF1	83	55	64	83	0.0063	0.7784
2	TATAAATW	TBP	32	106	17	130	0.0117	1
3	MGGAWGT	PEA3	72	66	55	92	0.0128	1
4	TnGCGTG	AHR	39	99	23	124	0.0142	1
5	WADTAAWTA	NKX6-2	53	85	36	111	0.0149	1
								
	*Top 1% Known Motifs Noncoding Strand*							
1	ABWCAGGTRnR	AREB6	13	125	2	145	0.0026	0.3205
2	KTWGTTT	SRY	91	47	71	76	0.0029	0.3487
3	ATTGTT	SOX-5	77	61	56	91	0.0030	0.3683
4	GCGCSAAA	E2F	0	138	8	139	0.0073	0.8725
5	RnCAGGTG	MYOD	68	70	50	97	0.0114	1
								
	*Top 1% Known Motifs Coding & Noncoding Strand*							
1	ABWCAGGTRnR	AREB6	25	113	6	141	0.0002	0.0240
2	CTTTGA	LEF1	108	30	96	51	0.0180	1
3	KTWGTTT	SRY	120	18	110	37	0.0107	1
4	ATTGTT	SOX-5	100	38	93	54	0.1014	1
5	RWAAACAA	FOXO1	96	42	83	64	0.0272	1
								
	*Top 5% Known Motifs Coding Strand*							
1	SCACGTG	MYC	141	594	190	576	0.0089	1
2	GnCnGTT	MYB	590	145	579	187	0.0296	1
3	RTGACTCAGCA	NF-E2	0	735	6	760	0.0311	1
4	MGGAWGT	PEA3	358	377	332	434	0.0384	1
5	KTWGTTT	SRY	473	262	454	312	0.0437	1
								
	*Top 5% Known Motifs Noncoding Strand*							
1	KTWGTTT	SRY	473	262	423	343	0.0003	0.0422
2	GCGCSAAA	E2F	13	722	31	735	0.0092	1
3	CYAATTWT	HOXA4	306	429	271	495	0.0146	1
4	TYAAGTG	NKX2-5	276	459	242	524	0.0168	1
5	GCCATnTT	YY1	168	567	137	629	0.0176	1
								
	*Top 5% Known Motifs Coding & Noncoding Strand*							
1	KTWGTTT	SRY	613	122	606	160	0.0346	1
2	WGATAR	GATA	691	44	698	68	0.0388	1
3	TYAAGTG	NKX2-5	450	285	420	346	0.0139	1
4	CCGGAART	ELK-1	97	638	69	697	0.0106	1
5	GTTRCYWnGYnAC	RFX1	12	723	4	762	0.0441	1

In addition to known sites, we sought to identify potential new regulatory motifs by examining the coding and noncoding strands with 174 previously identified phylogenetic motifs in the top 1% and 5% of our *S*+ vs. *S*- genes [28]. Eleven of these motifs represented palindromic sequences and were scanned in only the coding strand when both strands were analyzed. Again, while numerous motifs showed significance by Fisher's Exact testing (p < 0.05) only 1 survived multiple testing correction. Abbreviated results appear in Table 4. A single motif (CAGNYGKNAAA) showed a significant difference between the ER+ upregulated genes versus the ER- upregulated genes when the non-coding strand was examined in our top 1% gene list. Nineteen of 138 ER+ overexpressed genes contained at least 1 copy of the motif while only 3 of 147 genes contained the motif in the ER- overexpressed genes (adjusted p < 0.0373). This phylogenetic motif does not map to any known TFBS and represents a new target for exploration.

**Table 4:** Top Scoring Promoter Phylogenetic Motifs identified in Coding and Non-coding Strands. Top 1% and 5% Gene Sets.

**Rank**	**Phylogenetic Motif**	**Known Factor**	Position Bias*	**Genes with Motif**	**Genes w/o Motif**	**Genes with Motif**	**Genes w/o Motif**	**Fisher Raw p-value**	**Hochberg adj p-value**
				**Genes Overexpressed in ER+ Tumors**		**Genes Overexpressed in ER- Tumors**			

	*Top 1% Known Motifs Coding Strand*								
1	TCAnnTGAY	SREBP-1	-64	68	70	44	103	0.0010	0.1794
2	TAATTA	CHX10	-	70	68	46	101	0.0011	0.1895
3	RnTCAnnRnnYnATTW	-	-	15	123	3	144	0.0027	0.4563
4	CATTGTYY	SOX-9	-	30	108	13	134	0.0027	0.4690
5	CTTTGA	LEF1	-	83	55	64	83	0.0063	1
									
	*Top 1% Known Morifs Noncoding Strand*								
1	CAGnYGKnAAA	-	-	19	119	3	144	0.0002	0.0373
2	TAATTA	CHX10	-	70	68	46	101	0.0011	0.1895
3	TTAnWnAnTGGM	-	-	14	124	2	145	0.0014	0.2349
4	TTGTTT	FOXO4	-	98	40	79	68	0.0033	0.5672
5	YYCATTCAWW	POU1F1(*)	-	21	117	7	140	0.0046	0.7760
									
	*Top 1% Known Morifs Coding & Noncoding Strand*								
1	TAATTA	CHX10	-	70	68	46	101	0.0011	0.1906
2	CAGnYGKnAAA	-	-	26	112	9	138	0.0011	0.1918
3	TAAWWATAG	RSRFC4	-	31	107	14	133	0.0033	0.5631
4	TTGTTT	FOXO4	-	121	17	116	31	0.0574	1
5	CATTGTYY	SOX-9	-	45	93	27	120	0.0064	1
									
	*Top 5% Known Motifs Coding Strand*								
1	CTTTAAR	-	-	329	406	282	484	0.0019	0.3351
2	YKACATTT	-	-	174	561	133	633	0.0026	0.4526
3	TAATTA	CHX10	-	341	394	301	465	0.0057	0.9801
4	YATTnATC	CDP(*)	-	183	552	147	619	0.0088	1
5	TTGCWCAAY	C/EBPBETA	-	15	720	34	732	0.0090	1
									
	*Top 5% Known Morifs Noncoding Strand*								
1	WTGAAAT	-	-	307	428	263	503	0.0034	0.5955
2	TTGTTT	FOXO4	-	491	244	456	310	0.0039	0.6670
3	TAATTA	CHX10	-	341	394	301	465	0.0057	0.9801
4	YCATTAA	IPF1(*)	-	204	531	166	600	0.0070	1
5	TTAYRTAA	E4BP4	-	129	606	97	669	0.0093	1
									
	*Top 5% Known Morifs Coding & Noncoding Strand*								
1	TGCCAAR	NF-1	-	442	293	394	372	0.0007	0.1271
2	YCATTAA	IPF1(*)	-	326	409	282	484	0.0032	0.5575
3	YATGnWAAT	OCT-X	-	250	485	209	557	0.0051	0.8729
4	TAATTA	CHX10	-	341	394	301	465	0.0057	0.9744
5	AACYnnnnTTCCS	-	-53	76	659	49	717	0.0066	1

*Center of motif relative to transcriptional start site

### Analysis of the 3'UTR

We next screened for regulatory elements in the 3'UTR of our genes sets. Less is known about functional motifs in 3'UTRs than about functional motifs in promoter regions, but evolutionary conserved motifs in 3'UTRs may, as in promoter regions, indicate regulatory sites. We therefore used a previously identified set of evolutionary conserved 3'UTR motifs [28]. Although the function of half of these motifs is unknown, the remaining half has A/T rich elements believed to be involved in mRNA stability or represent likely microRNAs binding sites.

**Figure 2:**
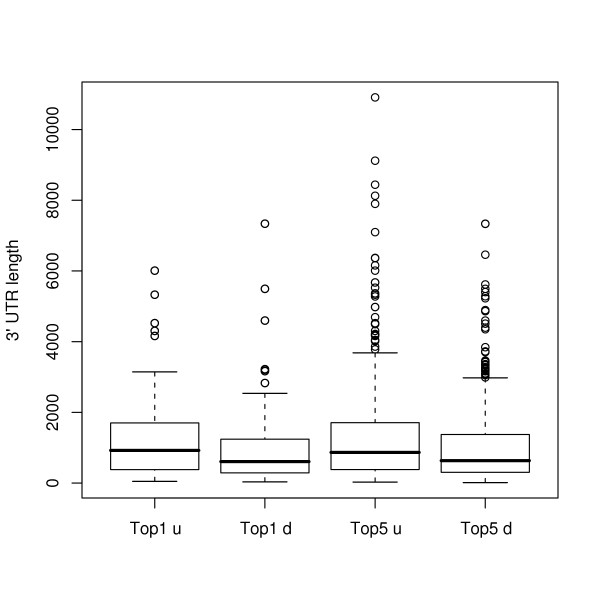
**Box plot of 3'UTR length differences**. Summary of ER+ upregulated genes ("Top1 u" and "Top5 u") and ER- upregulated genes ("Top1 d" and "Top5 d"). 3'UTR lengths were derived from RefSeq gene conversion as shown in Table 7.

We used the same RefSeq ID's to harvest the annotated 3'UTRs of our gene sets as described in the Methods. Surprisingly, we observed a significant difference in the median 3'UTR lengths between our gene sets (Figure 2). The top 1% genes overexpressed in ER+ tumors contained a median 3'UTR length of 0.9 kb, while genes overexpressed in ER- tumors contained a median 3'UTR length of 0.61 kb. A similar trend was observed when we examined the top 5% of genes sets. In this set, ER+ upregulated genes had a median UTR length of 0.87 kb while the ER- genes had a length of 0.63 kb. MicroRNA target genes have longer 3'UTRs, whereas anti-targets have shorter 3'UTRs [31]. Thus, the difference in 3'UTR length suggests a difference in miRNA targeting prevalence between the ER+ and ER- genes.

The most significant evolutionary conserved motif in the top 1% and top 5% genes (Table 5) correspond to a potential miRNA target site; YACTGCCR and WGCCTTA have seed complementarity to miR-34/miR-449 and miR-124. The miRNA seed region – nucleotides 2–8 from the 5' end – is the most important factor for miRNA target site recognition [32-34]. Fisher's Exact tests on the miRNA seed site occurrence counts, corrected for multiple testing, seemingly confirm that the ER+ genes are preferentially regulated by miRNAs, as all the significant seeds are overrepresented in the ER+ upregulated genes (Table 6). There is, however, a potential problem with using the Fisher's Exact test for the 3'UTR sets. If motif occurrences were random, we would expect the ER+ genes to have more motif occurrences than the ER- genes have, as the ER+ genes have longer 3'UTRs. Thus, to determine whether there is a significant difference in miRNA regulation between the ER+ and ER- genes, we had to address whether the occurrences of miRNA seed sites in the two sets were significantly different from what we would expect by chance. We therefore ran a set of randomization experiments where we compared the observed number of seed site occurrences in the ER+ and ER- genes' 3'UTRs with those in random gene sets that had 3'UTR lengths similar to the ER+ and ER- 3'UTRs (Table 6). We found that all of the seeds identified by significant Fisher's Exact tests do occur significantly more frequently in the ER+ 3'UTRs than in 3'UTRs from random gene sets. Moreover, these seeds also occur significantly less frequently in the ER- 3'UTRs than in random gene sets. Thus, it seems that whereas several miRNAs may coordinately regulate some of the ER+ genes, some of the ER- genes may collectively avoid being regulated by the same miRNAs.

**Table 5:** Top Scoring Phylogenetic 3'UTR Motifs Identified in 3'UTRs. Top 1% and 5% Gene Sets.

**Rank**	**Motif**	**Genes with Motif**	**Genes w/o Motif**	**Genes with Motif**	**Genes w/o Motif**	**Fisher's exact p-value**	**Hochberg adj p-value**
**Top 1% Phylo Motifs**		**Genes Overexpressed in ER+ Tumors**		**Genes Overexpressed in ER- Tumors**			

1	YACTGCCR	17	113	2	143	0.0002	0.0423
2	YYGCATGT	10	120	1	144	0.0037	1.0000
3	TGTANANAGA	12	118	3	142	0.0142	1.0000
4	TGCMNTAA	26	104	14	131	0.0168	1.0000
5	TGTGAA	51	79	37	108	0.0196	1.0000
6	TGTANNNTAG	13	117	4	141	0.0214	1.0000
7	TTTCTRNNAAA	2	128	11	134	0.0219	1.0000
8	AAGCACA	19	111	9	136	0.0273	1.0000
9	CTAKWTTT	23	107	12	133	0.0286	1.0000
10	TTTCTA	52	78	41	104	0.0423	1.0000

**Top 5% Phylo Motifs**							

1	WGCCTTA	134	562	80	652	< 0.0001	0.0031
2	CTAKWTTT	149	547	93	639	< 0.0001	0.0032
3	TGTGAA	305	391	238	494	< 0.0001	0.0034
4	TATATTT	210	486	149	583	< 0.0001	0.0066
5	TGTANNNTAG	73	623	36	696	0.0001	0.0235
6	TGTRNNNWATT	148	548	101	631	0.0002	0.0571
7	WRCCAAAA	113	583	71	661	0.0003	0.0706
8	TGTATANW	218	478	168	564	0.0004	0.1144
9	CTGTATWW	134	562	91	641	0.0005	0.1248
10	TGTRNTTT	310	386	261	471	0.0007	0.1747

**Table 6:** Top Scoring 6-mer and 7-mer miRNA seeds identified in normalized 3'UTRs. Top 1% and 5% Gene Sets.

**Rank**	**Seed**	**Fisher's exact p-value**	**Hochberg adj p-value**	**ER+ Randomiz. test p-val**	**ER- Randomiz. test p-val**	**Seed**	**Fisher's exact p-value**	**Hochberg adj p-value**	**ER+ Randomiz. test p-val**	**ER- Randomiz. test p-val**
Top 1% 6-mer						Top 1% 7-mer				

1	ATCTGG	0.0002	0.0547	0.000	0.018	CACTGCC	0.0011	0.4099	0.008	0.003
2	GGTACT	0.0005	0.1811	0.018	0.001	ACTATTA	0.0012	0.4328	0.000	0.019
3	AGCACA	0.0015	0.5048	0.005	0.004	TCTAGAG	0.0079	1.0000	0.021	0.006
4	CACTTT	0.0017	0.5921	0.001	0.029	ATTACAT	0.0113	1.0000	0.012	0.042
5	ACTGCC	0.0019	0.6339	0.004	0.028	GTCAACC	0.0146	1.0000	0.028	0.007
6	CTATTA	0.0025	0.8495	0.007	0.025	TGTATTA	0.0175	1.0000	0.052	0.022
7	AGTTTT	0.0035	1.0000	0.086	0.006	TGGTACT	0.0214	1.0000	0.007	0.117
8	GACACA	0.0059	1.0000	0.076	0.006	AAAGGGA	0.0228	1.0000	0.294	0.000
9	AGTCCA	0.0070	1.0000	0.027	0.006	AAGCACA	0.0273	1.0000	0.012	0.049
10	AGAGTT	0.0076	1.0000	0.218	0.001	GTGTTGA	0.0279	1.0000	0.462	0.000

Top 5% 6-mer						Top 5% 7-mer				

1	ATTATA	0.0000	0.0004	0.000	0.004	TGCCTTA	0.0000	0.0056	0.000	0.000
2	GCCTTA	0.0000	0.0031	0.000	0.002	ATATGCA	0.0000	0.0126	0.000	0.001
3	TGTTAA	0.0000	0.0038	0.000	0.000	TAATAAT	0.0003	0.1053	0.002	0.000
4	TTATAT	0.0000	0.0040	0.000	0.039	GATTTTT	0.0004	0.1569	0.000	0.008
5	TGAAGG	0.0000	0.0052	0.000	0.021	GTTATAT	0.0006	0.2226	0.000	0.004
6	TAAGCT	0.0000	0.0064	0.000	0.000	CCAACTC	0.0010	0.3524	0.015	0.000
7	ACTTCA	0.0000	0.0095	0.000	0.000	AATGCAT	0.0013	0.4888	0.000	0.008
8	ATTTCA	0.0000	0.0141	0.000	0.008	TCTGATA	0.0014	0.5104	0.140	0.000
9	CATTTG	0.0000	0.0150	0.000	0.014	ATTACAT	0.0014	0.5142	0.001	0.002
10	AGTATT	0.0001	0.0197	0.000	0.062	TCTGATC	0.0014	0.5186	0.000	0.107

Previous studies have identified several miRNAs that are aberrantly expressed in breast cancers [35,36]. Together the aberrantly expressed miRNAs in these studies mapped to 35 unique 6 mer seed sequences of which three were among our ten most significant 6 mer motifs. The three corresponding miRNAs (miR-205, miR-21, and miR-203) are all overexpressed in breast cancers. None of the ten most significant 6 mer motifs are from miRNAs reported to be differentially expressed in ER+ and ER- tumors [35]; the most significant 6 mer is ranked 25th (Hochberg-adjusted Fisher's Exact p-value of 0.17), is significantly more abundant in ER+ genes than expected by random, and is from miR-206, which is downregulated in ER+ tumors.

## Discussion

MicroRNAs are small (21–23 nucleotides) noncoding RNAs that recognize complementary target sequences in mRNAs and prompt either translational repression or RNA degradation. MicroRNAs play important roles in cancer. Iorio et al., for example, recently revealed that deregulation of multiple miRNAs can be correlated to pathogenic features such as estrogen or progesterone receptor status and tumor stage for breast cancers [35]. In addition, shorter postoperative survival times for patients with lung tumors can be predicted by measuring miRNA let-7 [37]. Thus miRNAs can be used both as classifiers of breast tumor type and as predictors of survival of lung cancer patients. MicroRNAs preferentially target 3'UTRs that have short sequences with perfect complementarity to nucleotides 2–7 (6 mer) or 2–8 (7 mer) in the miRNA's 5' region – the seed region [32-34]. As miRNA regulation may explain gene co-expression, we therefore included the 6 mer and 7 mer seed sequences for all human miRNA sequences known at the time of the study. We note that not all known human miRNAs are highly evolutionary conserved and these seed sequences therefore supplement the miRNA-related evolutionary conserved motifs.

Since we identified sets of genes that demonstrated differential expression between ER+ and ER- tumors, we reasoned that some of these genes may contain common *cis*-regulatory motifs contributing to their co-regulation. We would predict that these sites may, in some cases, be disproportionately represented between genes upregulated in ER+ tumors versus genes upregulated in ER- tumors perhaps allowing one to identify genes sharing common regulatory pathway. Computational tools exist to identify TFBS based upon over-representation of conserved motifs in datasets [38]. Other approaches aim to identify transcription factors (TF) which bind to TFBS based on the relatedness of expression profiles between the TF and the target genes they are postulated to regulate [39]. A combined approach utilizing expression measurements of tissue-specific gene sets in conjunction with orthologous TFs from humans and mouse provides for enhanced accuracy in predicting *bone fide cis*-regulatory elements [40]. For the most part these searches are guided by biologically confirmed TFBS interactions identified in the TRANSFAC database [41]; however, this approach may fail to identify motifs that may be evolutionarily conserved amongst mammals.

In addition to known sites that remained significant after multiple testing correction, many additional sites, and their associated transcription factors, warrant comment. A second important TFBS, CTTTGA, the binding site for lymphoid enhancer-binding factor 1 (LEF1), in the Top 1% Coding Strand ER+ overexpressed genes, failed rigorous multiple testing where 83 of 138 genes contained ≥ 1 site versus 64 of 147 genes in ER- gene set in Table 3. Nonetheless there is strong biological evidence supporting the role of LEF1 in tumorogenesis. The LEF1 binding site CTTTGA is one of the primary binding sites in the Wnt signaling pathway which regulates cell-cell adhesion and many morphogenetic events during mammary development and possibly cancer [42,43] Binding of Wnt proteins with frizzled protein prevents degradation of β-catenin, which subsequently translocates to the nucleus and binds transcription factors of the TCF/LEF family (this includes TCF8 discussed above and LEF1). Several tumors are known to have an altered β-catenin signaling pathway including colorectal and lymphoblastic tumors [44]. Mutations in the Wnt pathway genes can result in β-catenin stabilization and activation of LEF/TCF-induced transcription. Recent studies have demonstrated sebaceous tumors harboring *LEF1 *mutations interfere with β-catenin-binding domain of LEF1 and transcriptional activation [45]. Common human carcinomas also carry mutations in the β-catenin-binding domain of *LEF *[46]. Our data suggest that mutations (somatic or germline) in LEF1 or TCF8 binding sites in genes that inactivate Wnt signaling could contribute to breast tumorogenesis.

We did not find the estrogen receptor binding site (TGACCTTG) over-enriched in any our analyses. This is not surprising as our survey was confined to the immediate 2 kb promoter region. We point out that estrogen may be playing an indirect role on genes in ER+ overexpressing tumors via the activation of TF such as TCF8 which in turn activate downstream targets. Additionally, it is possible that differences in ER binding sites do exist between our gene sets but these sites may reside at distances much further upstream. Recent reports indicate that only two-thirds of ER TFBS can be localized to the proximal promoter region of RNA polymerase II genes [47]. We also note that the E2F binding site (GCGCSAAA) consistently ranked amongst the top 5 motifs (Table 3, 4^th ^highest scoring motif for top 1% and 2^nd ^highest scoring for top 5%) identified when screening the non-coding strand. In the non-coding strand of the top 1% gene sets, more E2F sites were observed in genes overexpressed in ER- tumors (8 of 147) versus 0 of 138 in genes overexpressed in ER+ tumors. Though the E2F site did not pass our multiple comparisons correction, published data support a role for these E2F sites in carcinogenesis. Prior efforts to identify a conditional regulatory program responsible for the coordinate regulation of sets of genes in multiple cancer types identified E2F as the lone TF universally overexpressed in multiple tumor types [48]. The presence of E2F sites exclusively in genes overexpressed in ER- BrCa tumors suggests that E2F plays a major role in this tumor type and may activate some target genes involved in cell cycle control [49].

A caveat to our analyses is the realization that in some cases the motif count alone may not be considered to be a good predictor due to positional bias of a given motif relative to the transcriptional start site (TSS). For some TFs, positional bias is likely to play a role in function. For example, the motif TATAAATW (TATA binding protein recognition sequence), well known for interactions with the basal transcription apparatus, shows a strong bias 23 bp upstream of the TSS. This spatial restriction is likely due to necessary interactions with the basal transcriptional apparatus (RNA Polymerase 2) [50]. Thus, motif copies present around -23 are likely to be functional while motifs distributed at other positions throughout the 2 kb upstream region would be predicted to be non-functional. Of our 174 phylogenetic motifs, only 32% (56 of 174) show positional bias, the majority of which are located within 100 bp of the TSS. The absence of any position bias for the vast majority of motifs in genes demonstrating disparate motif frequencies suggests a possible position-independent role in contributing to the observed expression patterns. The lone phylogenetic motif showing significance, CAGNYGKNAAA does not demonstrate positional bias.

A difficulty with any meta-analysis is that of study heterogeneity when one combines studies [51-53]. Meta-analyses on gene expression data are not immune from this criticism. There are many factors that influence a designation of ER+ and ER- status in breast tumors, including assay sensitivity and the scoring system used. The specific methods and assays for determining ER+ and ER- status are not available from Oncomine and we were unable to account for this factor in our results. Many have proposed statistical methods for quantifying the heterogeneity in a meta-analysis data set [54-56]. Since heterogeneity manifests in an inflation of inter-study variance, a meta-analysis with any degree of heterogeneity tends to bias the effect size toward the null hypothesis [57] and hence be conservative.

## Conclusion

Our meta-analysis was designed to identify genes showing consistent differences in gene expression patterns between ER+ versus ER- breast tumors. The target genes identified provide a unified set of genes obtained across multiple analyses and their expression patterns may reflect the true biological complexity of breast tumors. A small 10-gene meta-analysis signature to predict ER status has recently been described [13]. Three genes identified in their study (*ESR1*, *GATA3*, and *SLC39A6*) overlap with our top 1% ER+ upregulated genes. From our results, a more highly refined set of gene targets can potentially be explored that would prove useful in the development of an improved biomarker assays for determining not only ER status but also prognosis. Importantly, the overlap of 23 genes from our top 5% ER- upregulated tumors with a set of 69 genes demonstrating overexpression in more than 12 types of undifferentiated cancers via meta-profiling identifies genes universally activated in cancer. This list includes genes shown to be involved in the undifferentiated phenotype. They include the *MELK *kinase involved in mammalian embryogenesis, the apoptosis inhibitor *BIRC5*, and multiple genes implicated in cell cycle control (*CCNA2*, *MCM6 *and *FOXM1*).

By screening the proximal promoter and 3'UTR domains of our gene sets we wanted to identify both known TFBS, phylogenetically conserved motifs, and miRNA seed sequences that differ in prevalence between ER+ upregulated versus ER- upregulated genes. For any given site the disproportionate distribution between these gene sets may identify elements responsible for the co-regulation of groups of genes, and our analyses identified several significant elements in both the promoter and 3'UTR regions. Moreover, ER- genes had significantly shorter 3'UTRs than ER+ genes. Short 3'UTRs are common for miRNA anti-targets, which suggest that different mechanisms regulate groups of ER+ and ER- genes; that is, ER+ genes may be miRNA targets whereas ER- genes may be anti-targets. Consistent with this hypothesis, ER+ genes have significantly more putative miRNA target sites in common than expected by 3'UTR length alone, whereas ER- genes have significantly less putative miRNA target sites in common than expected by 3'UTR length alone. Anti-target genes are commonly involved in basic cellular processes [58] and in agreement with this, genes involved in the cell-cycle are significantly overrepresented in the ER- genes (data not shown).

Clearly, our analysis is a starting point. An examination of larger sequence domains upstream or these target genes may suggest additional elements showing differences in target abundance between these gene sets. While our phylogenetic motifs were for the most part small (<20 nucleotides), larger sequence elements such as enhancers that function at extended distances from these genes are likely to also play a role in the observed expression patterns. The potential importance of promoter motifs in gene expression and of common polymorphisms that reside within these sites was highlighted by a recent survey of the promoter regions of nearly 200 genes in which 75% of the SNPs identified modify (either by gain or loss) putative TFBS [59]. A survey of known polymorphisms (SNPs) from existing databases (dbSNP or HapMap) that reside within these motifs would also suggest the importance of these elements. It would be of keen interest to explore if regulatory modules exist within these gene sets consisting of combinations of both known and phylogenetically conserved motifs. Approaches such as this have been described computationally for yeast, fly, mouse and humans [60]. The recent use of comparative genomics tools from mammalian as well as evolutionarily distant species such as pufferfish (*Tetraodon *sp.) to identify phylogenetically conserved enhancers may also enable the identification of additional sequence elements responsible for the coordinate expression patterns seen for some of our genes [61]. Efforts such as these in conjunction with genomewide chromatin immunoprecipitation (ChIP) studies of promoter regions will provide a more comprehensive view of the key elements modulating the observed gene expression patterns.

Likewise, in the 3'UTR, genomewide efforts to map SNPs to miRNA target sites have revealed that many polymorphisms can either create new miRNA target sites or can lead to their loss [62]. Genome-wide searches in humans have identified *cis *polymorphisms in putative miRNA target sites that are likely contributors to phenotypic variation in humans and may to play a role in disease pathogenesis [63]. Future analyses will reveal whether SNPs in phylogenetically conserved promoter and 3'UTR elements can influence breast cancer risk at the level of RNA transcription or stability.

## Methods

### Meta-Analysis

We queried the Oncomine database [64] for gene expression studies in breast cancer as of September 2005. Within Oncomine, a dataset is considered to be "Analyzed" when the data from the original study is digitized and normalized into Oncomine's data mining system. At that time, there were 14 "Analyzed" studies with complete expression data in breast cancer. These "Analyzed" studies provided by Oncomine included normalized expression data per probe. Each probe's record included the probe's identification number (dependent on the array platform), the number of subjects, the mean expression values, and the p-value and q-value [65]. Although each study measured a variety of clinical aspects of patients with disease (e.g., progesterone receptor status, distant lymph node metastases, disease-free survival, etc.) 9 studies considered expression patterns between ER+ and ER- tumors. As estrogen receptor status is a key factor in treatment decision-making, we elected to compare the expression of genes overexpressed in ER+ tumors with those of ER- tumors. These 9 studies are listed in Table 1 and represent 954 independent cases of breast cancer.

For our meta-analysis we collected all of the expression data and imported all data sets into JMP tables for merging [66]. We considered Fisher's method for combining p-values as the basis of our meta-analysis statistic [67]. Rhodes et al [11] also considered this approach in their meta-analysis of gene expression in prostate cancer. Equation 1 shows our modification to the Fisher's statistic for our meta-analysis. Since we were not interested in the distributional properties of the Fisher's statistic, we modified the statistic by incorporating the signum of the direction of the differential gene expression. We mapped probe identifiers to unique UniGene IDs and these were the addends for *S *in Equation 1. However, if a study did not have a probe corresponding to a given UniGene ID, that study did not contribute to the meta-analysis statistic. Given that *m *studies have expression p-values for a given UniGene ID *p*_1_,..., *p*_*m*_, the meta-analysis statistic *S *is defined as

(1) $$S=-2\sum_{j=1}^{m}C_{j}\ln p_{j},$$

where *C*_*j *_= +1 if a given genes expression is higher in estrogen receptor negative (ER-) versus estrogen receptor positive (ER+) tumors while *C*_*j *_= -1 if a given gene's expression is higher in ER+ versus ER- tumors in any given study *j*.

Our convention for expression resulted in large negative values of *S *implying overexpression of genes associated with ER+ breast cancers while conversely large positive values of *S *indicated genes overexpressed in ER- breast tumors. Values of *S *close to zero imply neither over- nor underexpression of the gene. Herein, we will refer to "upregulated" and "downregulated" genes as those genes overexpressed in ER+ tumors, versus genes overexpressed in ER- tumors, respectively.

To compensate for the possibility that high values of *S *(either + or -) may be due to the contribution of high p-values from just a few studies rather than high p-values from multiple consistently significant studies, we normalized the *S *statistic by *N*, the number of studies in which a UniGene ID was present. The additional descriptive statistics that we considered for our meta-analysis included the number of studies that contained a probe for each UniGene ID, and the standard deviation (*SD*) of the (*C*_*j *_ln *p*_*j*_) addends of *S*. These statistics were used for summarization and discovery and not for consideration of any inferential or asymptotic statistical properties of *S*. We focused our subsequent analyses on a select set of genes by taking medians across each UniGene ID's *S*/*N *statistics. We selected sets of ER+ and ER- genes for further study by arbitrarily defining cutoffs at the upper 1% and 5% tails of the *S*/*N *distributions and including all genes with those *S*/*N *values or greater. We will refer to these as the "top 1% gene lists" and "top 5% gene lists" below. The complete list of Top 1% and 5% gene sets are in Additional File 1.

### Informatics

The difficulty of different gene annotation and naming conventions is well-known [68-71] and mandated that we select a common gene identifier. Since probes were dependent on both the array platform in the original studies, it was necessary to collapse the probes into one common identifier prior to our meta-analyses. We chose the UniGene nomenclature as a common identifier across all microarray probe sets. UniGene identifiers were chosen because each UniGene ID may capture multiple expressed sequence tags (ESTs) [72] on any given array. The lack of common probes or genes often occurs in array studies and is one possible explanation for the disparate gene sets identified between array studies [73]

We used the GEPAS' ID Converter batch formatting at the Bioinformatics Department at CIPF [74,75]. Owing to the diversity of probe nomenclature present on these arrays, our imported IDs included GenBank Accession numbers, clone IDs/IMAGE tags, and Affymetrix IDs. If a study's probe ID did not map to a UniGene ID, no information was contributed to the meta-analyzed expression value. In studies containing multiple probes for a given UniGene ID, each expression value was retained; we did not collapse nor statistically summarize expression values when multiple probes measured the same UniGene ID.

### Motif Screening

To screen for known motifs in the promoters of our ER+ and ER- gene classes we used a previously defined collapsed set of motifs from the TRANSFAC database v7.4 whereby highly redundant motifs were eliminated using weight matrix similarity as described in Xie et al. [28]. Xie et al. also identified conserved mammalian phylogenetic motifs in the promoter and 3'UTR domains [28]; these served as our reference motifs. MicroRNA 6-mer and 7-mer seed sequences corresponding to nucleotides 2–7 and 2–8 from the miRNA 5' end [34] were from miRBase release 9.1 [76]. We obtained RefSeq accession numbers mapping to each UniGene ID cluster by passing UniGene IDs from the top 1% and 5% gene lists through the conversion tool D.A.V.I.D [77]. RefSeq transcripts that were redundant as either duplicates or subsequences of other entries were removed. This removed redundancies that may unduly bias our motif comparisons, yet retained the sequences of as many transcripts as possible. RefSeq genomic intervals containing promoters and 3'UTRs were harvested from genomic resources (UCSC Genome Browser, NCBI Build 36.1). For each sequence list and each motif, a custom Python script counted the number of sequences with one or more motif occurrences within the set, and a Fisher exact test evaluated the significance of over or under representation in the ER+ versus the ER- sets.

For our promoter intervals some phylogenetic motifs represented sequences or subsequences of known TFBS while others were novel motifs having no known binding factors. For example, the phylogenetically conserved mammalian motif CAGGTG is a core subsequence for the E-box motif of helix-loop-helix TFs as well as the known binding site for the transcription factor MYC (S**CACGTG**). Alternatively, the phylogenetically conserved motif AGCYRWTTC does not represent any known TFBS. We limited our search to the proximal promoter space ranging from 2 kb 5' of the transcription start site (TSS) to 2 kb 3' downstream. If the translation start site was within 2 kb of the TSS site the shorter region was chosen so as to not overlap with the first coding exon. Collectively these promoter motifs ranged in length from 6–17 nucleotides. We separately screened the top one and five percent categories overexpressed in ER+ tumors (*S*-) and compared this to the same motif in genes overexpressed in the top one and five percent of ER- tumors (*S+*) respectively.

Table 7 shows the results for our UniGene ID conversion, number of RefSeq mRNAs identified, and the final number of RefSeq mRNAs after subsequence filtering. Though our initial analysis returned more RefSeq mRNAs than input UniGene IDs, after subsequence filtering the yield of RefSeq mRNAs ranged from 77–98%. Collectively, we feel it represents a balanced collection of unique RefSeq IDs minimizing transcript redundancy yet faithfully representing the transcript diversity observed in our meta-analysis. For promoter analyses we surveyed both coding and non-coding strands as this provided a comprehensive survey of the motif distribution since earlier work suggests functional TFBS may be independent of strand orientation [78]. Additionally, we elected to survey the entirety of the sequence space without filtering repeat elements as previous studies demonstrate that TFBS sites may reside in these elements [79]. For palindromic motifs we only screened the coding strand in our promoter survey.

**Table 7:** Conversion of UniGene IDs to RefSeq mRNAs utilizing D.A.V.I.D.

**The number of promoter sequences analyzed**				
Step	Top 1% S-	Top 1% S+	Top 5% S-	Top 5% S+
UniGene	150	150	902	902
RefSeq mapped	168	192	1072	1116
RefSeq downloaded	167	167	850	888
RefSeq unique	138	147	735	766

**The number of 3'UTR sequences analyzed**				

Step	Top 1% S-	Top 1% S+	Top 5% S-	Top 5% S+
UniGene	150	150	902	902
RefSeq mapped	168	192	1072	1116
RefSeq downloaded	165	166	844	887
RefSeq unique	130	145	696	732

S-, genes overexpressed in ER+ tumors; S+, genes overexpressed in ER- tumors

## Abbreviations

BrCa: breast cancer; ER+ and ER-: estrogen receptor positive and negative breast cancer, respectively; 3'UTR: 3' untranslated region; TF: transcription factor; TFBS: transcription factor binding site; miRNA: microRNA; CRM: *cis*-regulatory modules; ESTs: expressed sequence tags; TSS: transcription start site; Oncomine DB: Oncomine database; ChIP: chromatin immunoprecipitation; *SD*: standard deviation; *S+ *and *S*-: list of genes overexpressed in ER- tumors and ER+ tumors.

## Authors' contributions

DDS extracted data from Oncomine, performed the statistical analyses and drafted the manuscript. PS programmed the 3'UTR and promoter parsers and performed the randomization tests. OS conceived of the 3'UTR design and subsequent analysis. CL and GER extracted data from Oncomine and performed the Ingenuity analysis. CG edited the manuscript and contributed to the motif discovery portions. GL designed the project, provided overall guidance, edited and drafted the manuscript. All authors read and approved the final manuscript.

## Supplementary Material

Additional file 1 Top 1% and Top 5% Gene Sets from Meta-Analysis. The data provided represents the top 1% and 5% up- and down-regulated genes among the studies considered in our meta-analysis. The data are sorted by S/N, which is analogous to an estimate of average expression per study. Click here for file
